# Preputial Deformities a Source of Serious Nervous Disturbances

**Published:** 1881-08

**Authors:** R. W. Westmoreland

**Affiliations:** Atlanta, Ga.


### PREPUTIAL DEFORMITIES—A SOURCE OF SERIOUS NERVOUS DISTURBANCES.

By ROBERT W. WESTMORELAND, M.D., Atlanta, Ga.

In considering the connection or pathological sequence of preputial diseases, and different centric derangements of the nervous centres, we are often surprised at the apparant insignificance of the cause in comparison with the ultra effect. For this one reason there are many skeptics on this subject, and some who are so extremely skeptical that they will insist on the incorrectness of attributing any pronounced nervous disturbances to even an extreme preputial adhesion or contraction. It is not strange, however, that this should be the case, since expert observers and scientific experimenters are not wanting who deny that such serious organic lesions as paralysis, for instance, may result secondarily, through reflex agencies, from any form of genito-urinary irritation. This tendency, among neurologists, to deny the accuracy of the doctrine of the reflex agency in some of the worst forms of nervous derangement, has been on the increase for several years, and many theories are advanced in lieu of the reflex ones which are sustained by laborious and patient observation and experimentation. The result of this move has, no doubt, been to restrain the too free and general use of the doctrine in diagnosing many diseases, but, at the same time, "there is no doubt that in a few cases the theory of reflex inhibitory action is alone sufficient to account for" the many phenomena of nervous derangement observed. In confirmation of this view the case of paralysis reported by Landy may be cited, in which cure resulted in the correction of an associated anteversion of the uterus. Rosenthal relieved, entirely, a case of paraparesis by extracting a needle which had been introduced into the vagina. Madge and Fuller likewise report cases illustrative of the same principle.

As in instances where post mortems have been available, localized inflammation of the cord or its meninges have been observed, the antagonists of the reflex doctrine instituted the opposing one of *neuritis migrans.* This consists in the theory that peripheric disturbance must amount to inflammation to produce the centric derangement which, by extension, assumes in the centre the primary affection of the periphery. This argument would seem very specious, and, were the morbid anatomy presented on an autopsical examination the only evidence, conclusive. Certain physiological, combined with other pathological facts, however, go far toward the demonstration of a contrary position.

Stimuli, as the invariable originators of the manifestation of nerve force, operate through their peculiar vital influence on the peripheral origins of the afferent and upon the central origins of the efferent nerves. Morbid variations of this stimulus, at the periphery, produce corresponding alterations at the centre, and following a well known law, a progressive and continued morbid excitation results in an equally morbid degree of depression or debility.

The injurious influence of continued and progressive excitants applied to nerve tissue has the effect of weakening, and finally of exhausting its power of responding. Repose from this stimulation is the only remedy, and without this so far from the benefits of the necessary processes of repair taking place, the progressive degenerative and retrograde influences assert themselves. And further, the physiological effect of any continued stimulation would be an increased circulation in the part stimulated. An increase in the circulation of any part from which afferent nerves arises increases their readiness to receive impressions, and in that way would be affected a proportional increase of the functional activity of the receiving centre.

That extreme cases of irritation, resulting in reflex derangements exhibit no perceptible increase of local sensation is accounted for by the physiological fact that there is no specific relation between reflex action and sensory activity; the slightest irritation producing more decided reflex phenomena than the most painful application. From these considerations we are led to conclude that a certain class of spinal troubles may, and do, originate from peripheral irritation, and we feel assured that clinical observation will bear out this conclusion. On this account in all troubles of a nervous character, where it is difficult to assign any certain cause otherwise, examine for this peripheral irritation, and, very often, the problem will be solved. In young childen, particularly boys, this will be found essential in making a correct diagnosis. While the genitals may oftener be the seat of difficulty, still it will be found the rectum and other parts than the prepuce of the genito-urinary apparatus will be found the point of origin. We have had occasion to observe the ill affects of failure to recognize the importance of this fact in diagnosing, and have seen patients treated for almost every thing, when by a simple surgical operation more good might be done than by the whole materia medica. Very recently we were called upon to treat a babe, about one year old, for paralysis. The parents represented that this condition had been progressing until an utter impossibility to stand up had occurred. The child had been treated for teething and many other troubles, with no benefit. Upon examination of the genitals I found phimosis and an entire adhesion of the prepuce to the whole glands, extending up to the very border of the meatus. This condition was corrected by incising the prepuce, and breaking up the adhesion. Time sufficient to indicate any great change has not elapsed, but we feel confident that decided benefit will result from the operation. It may be well to state that in the foregoing case excessive perspiration and incontinence of urine were prominent attending symptoms, and were decidedly relieved by the operation, especially the former.

A contraction of the meatus urinarius is another prolific source of serious centric disturbances, but its effect in this way is as much attributed to the connection with vesical or urethral irritation, which is a usual sequence of stricture of this outlet, as to the contraction itself.

It would be well, therefore, we think, in all cases of nervous disease from uncertain causes to search diligently for these peripheral irritations, and when found, relieve or correct them. Many apparently hopeless cases will, by these means, be entirely cured, and all of them be at least benefitted. Particularly in the case of children is this true. The majority of males are born with more or less decided preputial imperfections. Many of these being permanent, and acting, probably, upon a naturally sensitive nervous organization, finally establish an extreme centric derangement.

Most usually some form of vesical irregularity will point to the primary seat of difficulty, but an ocular examination will always settle the question. Whether it be the adherent or contracted prepuce or occluded meatus causing the difficulty, the indication is always to operate.

For adherent prepuce, it is necessary to break up the adhesion and interpose greased lint between the raw surfaces until healing takes place. If a contracted condition, in addition to the adhesion of the prepuce exist incision or circumcision, preferably the latter, should be practiced.

Where a constricted meatus is the prime source of reflex disturbances, a free incision in the floor extremity of the orifice, the surfaces being kept apart by greased pledgets of lint, will be of much benefit.

It is not the object of this paper to discuss the operative influences of stricture, irritable bladder, uterine or rectal diseases instituting reflex disturbances, but to merely direct attention to the importance of regarding the principle that peripheric irritation may excite grave centric disturbances, and that where idiopathic disease of a nerve centre cannot be substantiated, we may beneficially refer to disturbances at the periphrey.

